# The Construction and Initial Application of Chinese College Students’ Epistemological Beliefs Questionnaire

**DOI:** 10.3389/fpsyg.2020.00054

**Published:** 2020-01-29

**Authors:** Yan Zhou, Dingliang Tan

**Affiliations:** ^1^Department of Psychology, School of Education Science, Liaocheng University, Liaocheng, China; ^2^School of Education Science, Nanjing Normal University, Nanjing, China

**Keywords:** college students, epistemological beliefs, questionnaire construct, developmental characteristics, Chinese

## Abstract

The authors construct Chinese college students’ epistemological beliefs questionnaire, which consists of six dimensions: certain knowledge, simple knowledge, acquisition of knowledge, ability to learn, speed of learning and the value of learning. The questionnaire has good reliability and validity. Using this questionnaire, based on the investigation of 1121 college students from eight different universities in China, this study aims to explore the developmental characteristics of Chinese students’ epistemological beliefs. Results show that the development of college students’ epistemological beliefs follows an increased pattern; the scores of the junior and senior students are higher than the freshman and sophomore, and the scores of the sophomore are the lowest in these four groups. College students’ development on the dimensions of knowledge beliefs is higher than that of learning beliefs. The investigation and interview suggest that Chinese college students’ epistemological beliefs experience three stages: the first stage is multiplicity, the second stage is primary relativism and the third stage is advanced relativism.

## Introduction

### The Research Tool of College Students’ Epistemological Beliefs

Beliefs about knowledge and knowing, namely epistemological beliefs are individuals’ representations about the nature, organization, and source of knowledge, its truth value and the justification criteria of assertions (Hofer and Pintrich, 1997, 2002). Chinese scholars maintain that it includes the attitude and evaluation of knowledge, learning experience, learning phenomenon, and so on (Zhou and Tan, 2016). Because students’ epistemological beliefs have great impact on their learning process and learning outcomes (Hofer, 2004; Schraw and Sinatra, 2004; Muis et al., 2006; Bromme et al., 2010; Braten et al., 2011), the researches of epistemological beliefs have become hot issues of educational psychologist (Buehl and Alexander, 2001; Hofer, 2001; Watkins, 2004).

The research tool of epistemological beliefs is Schommer’s Epistemological Questionnaire (SEQ). This questionnaire consists of four dimensions: certain knowledge, simple knowledge, innate ability, and quick learning (Schommer, 1990). When researchers use SEQ in Asian countries, they found some different dimensions and low reliability and validity about SEQ. These differences can be accounted for in terms of differences in cultural context. For example, Chan and Elliott use SEQ in Hong Kong students, but only three factors are extracted from SEQ. Two factors (Authority-expert knowledge and learning effort-process) are different from SEQ; these reflect the unique characteristics of Hong Kong students’ epistemological beliefs. Furthermore, a reliability check of SEQ yielded low to moderate Cronbach alpha values, ranging from 0.1 to 0.58. These data cast doubt on the reliability and validity of the scale and also on the general applicability of SEQ across cultural groups (Chan and Elliott, 2002). The study conducted by Wang showed: three factors are extracted from SEQ when it is applied to Chinese middle school students, only the factor of simple knowledge is consistent with Schommer’s research, two factors (innate ability, quick learning) are not extracted. Furthermore, Cronbach alpha values of factor 2 and factor 3 are low, the coefficients are 0.2119 and 0.4570, split-half coefficients are 0.1516 and 0.5588 (Wang, 2004). The results show that it is unsuitable to apply SEQ directly in Chinese culture. It is necessary to construct a native epistemological questionnaire which can be used in Chinese context.

### The Developmental Characteristics of Epistemological Beliefs

How do individual epistemological beliefs develop with the growth of age and educational level? It is one of the hottest issues in epistemological researches. Many scholars proposed diversiform epistemological beliefs developmental model. For example, Perry’s Scheme of Intellectual and Ethnical Development included a sequence of nine positions: basic duality, full dualism, early multiplicity, later multiplicity, contextual relativism, pre-commitment, commitment, challenges to commitment and post- commitment. The nine positions of the scheme have typically been clustered into four sequential categories: dualism (including positions 1 and 2), multiplicity (including positions 3 and 4), relativism (including positions 5 and 6), and commitment within relativism (including position 7 through 9) (Hofer and Pintrich, 1997). Within Baxter Magolda’s Epistemological Reflection Model, there are four different epistemological assumptions: absolute knower, transitional knower, independent knower, contextual knower (Hofer and Pintrich, 1997). The Reflective Judgment Model proposed by King and Kitchener consists of seven qualitatively different stages that describe how individuals epistemological beliefs development. Within the seven-stage model there are three levels: pre-reflective (stages 1, 2, and 3), quasi-reflective (stages 4 and 5) and reflective (stages 6 and 7) (King and Kitchener, 2002). Kuhn defines four categories of epistemological views in her Argumentative Reasoning Model: realist, absolutist, multiplist and evaluativist (Kuhn et al., 2000). These models mentioned above have same view that epistemological beliefs follows a general stage sequence through their presentation are different. For example, Perry’s dualism stage, Magolda’s absolute knower and Kuhu’s absolutist are in accordance with King’s pre-reflective; while King’s quasi-reflective is a level between Perry’s multiplicity and relativism; Magolda’s contextual knower is corresponding to King’s reflective level.

In addition to the developmental research mentioned above, the scholars who suggest that epistemological beliefs are multidimensional systems also explore the characteristics. In a study conducted by Schommer, SEQ was administered to students. The results showed: Belief in simple knowledge, certain knowledge, and quick learning decreased from freshman to senior year (Schommer, 1993b). The epistemological beliefs may not develop in synchrony. Some dimensions are considered mature; meanwhile other dimensions are naive (Schommer, 1993a; Schommer-Aikins, 2004).

Epistemological beliefs are influenced by socio-cultural factors (Hofer, 2008; Khine, 2008; Tabak and Weinstock, 2008). Do the models suggested by western scholars adapt to Chinese students? Are there any different characteristics between the western students and Chinese students who are living in the circle of Confucius culture? There are no clear answers. Therefore, this study aims to explore the developmental trend of Chinese college students’ epistemological beliefs.

## Materials and Methods

### Sample

The sample consists of 1300 college students from eight universities: Nanjing Normal University, East China University of Petroleum, Nanjing Xiaozhuang University, Nantong University, Liaocheng University, Qingdao Agricultural University, Zhejiang Agricultural and Forestry University, Weifang Medical University. The classification of specialty includes arts, science, medical, etc. Finally, 1121 students’ questionnaires are selected; the effective percentage is 86.23%. The age ranges from 17 to 24, and the mean of the age is 21.06 (*SD* = 1.36). The valid questionnaires are split into two parts randomly, one part of 560 questionnaires is used for exploratory factor analysis and another part of 561 questionnaires for confirmatory factor analysis. 1121 questionnaires are used to the data analysis of developmental research.

### Data Analysis

SPSS16.0 and AMOS16.0 were used to process the data.

## Results

### The Construction of College Students’ Epistemological Beliefs Questionnaire

#### Items Construction

When constructing the questionnaire, we combined literature analysis, interview and investigation research. First, many documents and questionnaires on epistemological beliefs were consulted, such as: Schommer’s SEQ; Schraw’ EBI; epistemological beliefs questionnaire compiled by Chan and Elliott. Then semi-opened interviews with some college students were carried out. The interview questions are mainly about the beliefs about knowledge and learning, such as “Where does knowledge come from, books or individual construction?,” “Is the knowledge in the textbooks accurate?,” “What is the judging standards of knowledge acquired?,” “Is the ability to acquire knowledge innate or learned?,” “Do you agree that some students are just born smart, others are born dumb?,” “Do you like science research?,” and “What is your emotional experience in learning, happy or boring?”

Related items are collected through interviews and other epistemological beliefs questionnaires, so as to work out predicting items for Chinese students’ epistemological questionnaire. Finally, 89 items are screened out and used to construct the preliminary questionnaire.

One class of students is randomly selected from Nanjing Normal University, they are required to find out the items difficult to understand or with unclear explanation. Meanwhile, the preliminary questionnaire is modified with the assistance of two psychologists and six college teachers, in order to make it understandable and suitable for college students. Based on those preparations, some items are deleted and modified. Finally, 81 items are determined to compose the questionnaire.

#### Item Analysis

First, the discrimination index of every item is calculated, respectively. Taking discrimination index of less than 0.30 as criteria in item analysis, item 39 is deleted, because the results show its discrimination index is 0.07.

Second, the critical ratio of every item is calculated, respectively. The results show all the items’ critical ratio is significant (*P* < 0.05). Critical ratio more than 3 is taken as strict criteria in this research, as scholar suggests that when the critical ratio is more than 3, the item will have good discriminability (Qiu, 2002). According to this criteria, item 81 is deleted (*t*=2.096, *P* = 0.038).

#### Exploratory Factor Analysis

The sampling data is subjected to Kaiser-Meyer-Olkin test and Bartlett spherical test. Parameter for sampling propriety KMO in exploratory factor analysis is 0.848, the statistics of Bartlett spherical test is 13440 (*df* = 3081, *P* < 0.001), suggesting that the data is suitable for factor analysis.

Method of principal component analysis is used to extract the factors, and oblique rotation method is used to calculate the final factor-loading matrix. Step exclusive method is used to delete items, and the items with low factor-load (less than 0.40), low generality and low total factor-loading (less than 0.35) are removed. The number of factors is decided based on the following criteria: (1) the eigenvalue of each factor is over 1; (2) factor solution should accord with Scree Test; (3) selected factors can explain no less than 3% of the total variance before rotation; and (4) each factor consists of more than 3 items. In addition, questions are screened out according to the former founded theoretical structure. Finally, exploratory factor analysis for the questionnaire selects 38 items, and extracts six clear factors. 52.39% of the total variances can be explained.

The results of exploratory factor analysis show: 38 items have high factor-loading at their factor. The lowest loading is 0.423 and the highest loading is 0.765. Generality of items ranges from 0.34 to 0.74. The eigenvalue of six factors are 3.879, 3.183, 2.593, 2.331, 2.301, and 1.793; and 15.463%, 10.208%, 8.376%, 6.824%, 6.135%, and 4.719% of the variances can be explained, respectively. The accumulative contribution rate is more than 50%, indicating that these 38 items are good indicators of the six dimensions and the questionnaire has good structure validity.

The questionnaire consists of six dimensions, which are named according to the theoretical conception of the model and the specific meaning of each item. The first dimension, termed as “acquisition of knowledge,” includes 9 items, and concerns the students’ beliefs about the source of knowledge and about the judging standards of whether the knowledge is acquired or not. The second dimension, termed as “value of learning,” includes 8 items and deals with the beliefs of the value of knowledge and learning. The third dimension, termed as “speed of learning,” includes 6 items and examines the beliefs of learning speed. The fourth dimension, termed as “certain knowledge,” includes 5 items and concerns the students’ beliefs about the development of knowledge, aiming to explore whether the knowledge is evolving or fixed and determined. The fifth dimension termed as “ability to learn,” includes 6 items and deals with the beliefs of the ability to learn, aiming to know whether it is innate or learned. The sixth dimension, termed as “simple knowledge,” includes 4 items and examines the students’ view of the simplicity and connection of knowledge, and whether knowledge is isolated or closely linked with each other. The questionnaire could be seen in the Appendix.

#### Confirmatory Factor Analysis

**FIGURE 1:**
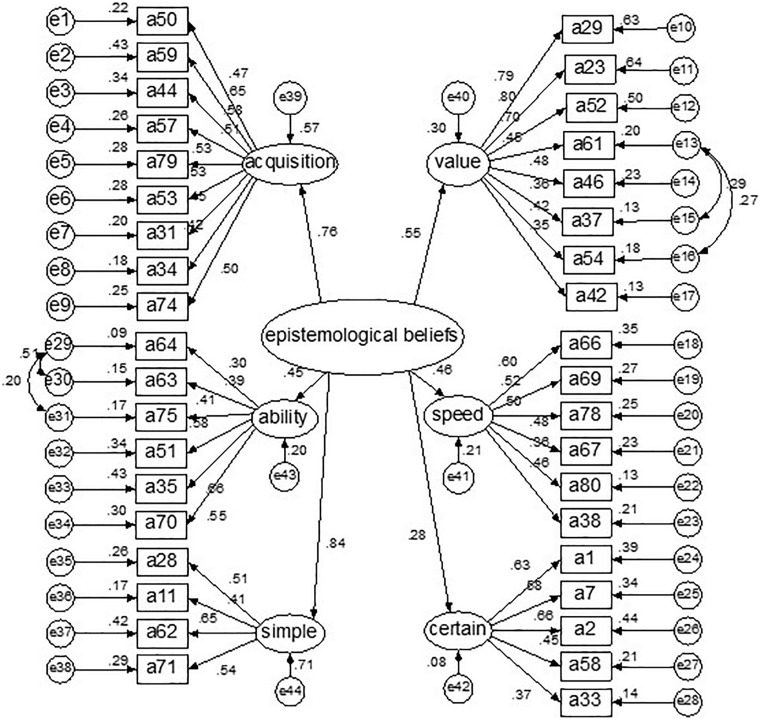
CFA results of Chinese college students’ epistemological beliefs model.

In this research, the hypothesized model is proposed: Six related factors (certain knowledge, simple knowledge, acquisition of knowledge, ability to learn, speed of learning, the value of learning) are established parallel. And the six factors belong to a second-level – epistemological beliefs. The model is shown in Figure 1.

Maximum likelihood method is used to analyze the data; the goodness of fits of the model is as follows: χ2/*df* = *2.412, GFI* = *0.905, AGFI* = *0.889, CFI* = *0.837, IFI* = *0.809, NNFI* = *0.815, and RMSEA* = *0.050.*

#### Reliability Analysis

Cronbach alpha is used to determine the reliability of the questionnaire. The Cronbach’s α of the whole questionnaire is 0.830, and the Cronbach’s α of the knowledge beliefs subscale (includes acquisition of knowledge, certain knowledge and simple knowledge) and the learning beliefs subscale (includes value of learning, speed of learning and ability to learn) are 0.801 and 0.796, respectively. The Cronbach’s α of the six dimensions ranges 0.609 to 0.766. The reliability of the whole questionnaire and dimension is considered as acceptable.

### The Developmental Characteristics of College Students’ Epistemological Beliefs

**FIGURE 2:**
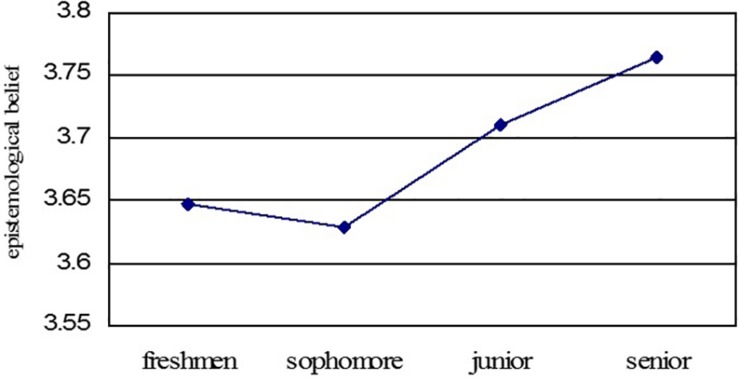
The general development trend of college students’ epistemological beliefs.

**FIGURE 3:**
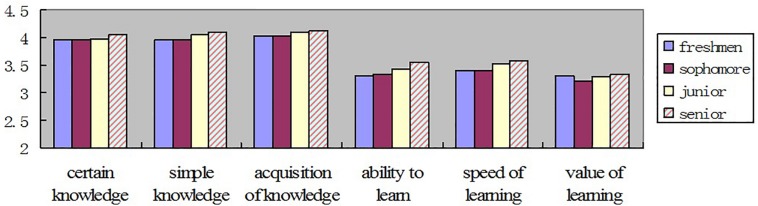
The development of the dimensions on college students’ epistemological beliefs among different grades.

One-way Anova is used to compare the grade differences on college students’ epistemological beliefs. The result shows that there are significant differences among grades (*F* = 8.782, *P* < 0.001). The general development trend of college students’ epistemological beliefs follows a raised pattern; the scores of the junior (*M* = 3.710) and the senior (*M* = 3.764) are higher than that of the freshman (*M* = 3.647) and the sophomore (*M* = 3.629), and the scores of the sophomore are the lowest in these four grades. The general development trend is shown in Figure 2, and the specific development of each dimension is shown in Figure 3.

As is shown in Figure 3, the scores of the three dimensions of knowledge beliefs are higher than the three dimensions of learning beliefs. It indicates that college students’ development on the dimensions of knowledge beliefs is higher than that of learning beliefs.

## Discussion

### The Construction of College Students’ Epistemological Beliefs Questionnaire

Based on the interview and other epistemological beliefs questionnaire, a predicting questionnaire is worked out. Modified with the assistance of psychologists, the preliminary questionnaire has good content validity. Then the item analysis and exploratory factor analysis are used to select items, and the method of confirmatory factor analysis is used to test its structural validity. Finally, the internal consistency is used to evaluate the reliability of the questionnaire. The results show that the questionnaire has good reliability and validity and can be used to investigate Chinese college students’ epistemological beliefs. Comparing previous models, our model was constructed based on the investigations of Chinese college students, while the model from Wang (2004) based on Chinese middle school students and SEQ based on America students. Considering the sample difference and psychometric indicators, our model is suitable to representate Chinese college students’ epistemological beliefs.

### The Developmental Characteristics of College Students’ Epistemological Beliefs

The general development of college students’ epistemological beliefs follows a raised pattern. The scores of the sophomore are the lowest in these four grades, and we consider it is related to the Chinese students’ learning environment. Freshmen go into colleges after hard working at middle schools, thus their beliefs about knowledge and learning are still continuous with their beliefs at middle schools. Through the preparing process of college entrance examination, they have obtained profound experience on the relation and the organization of knowledge and the effect of hard work. The positive reinforcement of having passed the college entrance examination forges the students hold a positive attitude toward the learning ability and its value. However, their learning styles have to be adjusted because of the big differences of the teaching styles and management modes of the college teachers from that of middle school teachers. The adjustment process wavered their original epistemological beliefs, whereas the new effective epistemological beliefs system has not been founded, which will result in the transitory low ebb stage. The epistemological beliefs level of the junior and the senior have been promoted with the accumulation of knowledge and learning experience after they spending the fresh stage of the 1st year and the confusion stage of the 2nd year in college.

The investigation and interview suggest that Chinese college students’ epistemological beliefs experience three stages.

The first stage is multiplicity, which is a stage as Perry’s later multiplicity, Magolda’s transitional knower, King and Kitchener’s stage 4 in quasi-reflective and Kuhu’s multiplist. With the cognition of diversity and uncertainty of knowledge, the individual in this stage is inclined to believe that each person has a right to his or her own opinion. Beliefs are justified by giving reasons and using evidence, but the importance of assessment reasoning is still obscure. Although most students realize the learning process is constructive, they still follow authorities blindly. The majority of freshmen and sophomores are at this stage.

The second stage is primary relativism, which is a stage as Perry’s contextual relativism, Magolda’s independent knower, King and Kitchener’s stage 5 in quasi-reflective and Kuhu’s evaluativist. Students in this stage think that knowledge is contextual and subjective since it is filtered through a person’s perceptions and criteria for judgment. Beliefs are justified within a particular context by means of the rules of inquiry for that context and by context-specific interpretations of evidence. Students in this stage have seen the necessity of making choices and committing a solution. They realized that all proposed solutions are supported by reasons and must be viewed in context to support. Students’ task is not only to memorize and duplicate knowledge, but to learn the way of thinking and making critical judgments. Sometimes students in this stage will give up their thinking or reflection because of the lacking of the skill of rational criticism, and their epistemological beliefs are not mature totally. Most of the juniors and seniors are at this relativism stage.

The third stage is advanced relativism, which is a stage in accordance with Perry’s pre-commitment and commitment, Magolda’s contextual knower, King and Kitchener’s stage 6 in reflective thinking. Students in this stage realize that the source of knowledge is from the inner individual construction instead of the outer authority. Integration occurs of the knowledge learned from others with those from personal experience and reflections. Beliefs are justified by comparing evidence and opinion from different perspectives on an issue or across different contests and by constructing solutions that are evaluated by criteria such as the weight of the evidence. AS King and Kitchener pointed out: “*One can judge an argument by how well thought-out the positions are, what kinds of reasoning and evidence are used to support it, and how consistent the way one argues on this topic is as compared with other topics*” (King and Kitchener, 1994). Some of the juniors and seniors are at this stage.

As to the six dimensions, the scores of the three dimensions of knowledge are higher than the three dimensions of learning; it indicates that the knowledge developmental level is higher than the learning developmental level. With abundant learning experiences, college students realize they must be active in learning process, and they gradually have formed deep comprehension about the process of knowledge acquisition. As the college students can have enough time to learn the knowledge which they are interested in, and there are a variety of courses are offered, which makes them hold mature view on the connection and development of knowledge. Compared with the knowledge dimensions, the beliefs about learning ability and speed are not so closed with the abundant learning experiences and variety of courses. Especially in the aspects of the beliefs about learning ability, students often draw conclusion through the comparison with students around them. However, the differences of learning ability among college students shrink after they passed the college entrance examination. Those students who have got high scores in middle schools can not promise to be excellent in college studies all the time. So some students will have surface awareness of the indigenous development of learning ability. Meanwhile, the beliefs about the value of learning are mostly connected with social environments. Nowadays, faced with employment pressure, college students have a dim consciousness of the learning value. Moreover, less reflection on the epistemological questions such as the learning speed leads to college students getting lower scores on learning beliefs than that on knowledge beliefs. The results show that the dimensions of epistemological beliefs are asynchronous developed, which is in accordance with Schommer’s opinions (Schommer, 1993a; Schommer-Aikins, 2004).

For a correct interpretation of our results, it is necessary to consider its limitations. The results are based upon a norming sample of students in Eastern China, perhaps it is feasible that students attending universities in other Asian culture may not show the same pattern. This study did not explore other college students’ in other areas due to sampling limitations, which may limit the generalizability of the findings of our study.

## Conclusion

College students’ epistemological beliefs questionnaire includes 38 items and consists of six dimensions: certain knowledge, simple knowledge, acquisition of knowledge, ability to learn, speed of learning, and the value of learning. The questionnaire has good reliability and validity and can be used to investigate Chinese college students’ epistemological beliefs.

The development of college students’ epistemological beliefs follows an increased pattern; the scores of the junior and the senior students are higher than the freshman and sophomore, and the scores of the sophomore are the lowest in these four grades. The knowledge developmental level is higher than the learning developmental level.

## Data Availability Statement

The datasets generated for this study are available on request to the corresponding author.

## Ethics Statement

The studies involving human participants were reviewed and approved by the ethics committee of Liaocheng University. The patients/participants provided their written informed consent to participate in this study. Participants were allowed to withdraw from the study whenever they wanted and the data were collected anonymously.

## Author Contributions

YZ designed the investigation, collected the data, analyzed the data, and wrote the manuscript. DT provided theoretical guidance and generated items.

## Conflict of Interest

The authors declare that the research was conducted in the absence of any commercial or financial relationships that could be construed as a potential conflict of interest.

## Appendix

### Chinese College Students’ Epistemological Beliefs Questionnaire

#### Certain Knowledge

Knowledge that is considered correct today may change tomorrow.
What you have learned now will need to be adjusted due to time or other reasons.
Knowledge may be modified after a certain period of time.
Knowledge is uncertain. It changes over time.
What is true today may become false tomorrow.

#### Simple Knowledge

The knowledge of each course is closely linked.
Most knowledge is broadly linked.
Knowledge is closely related to the real world.
Each discipline has its own set of knowledge, which has little relation to each other.^∗^

#### Acquisition of Knowledge

The key to acquiring knowledge is to study and comprehend by oneself.
How much we gain in learning depends on our own initiative.
The key to how much students can learn in class is their own understanding of what they have learned.
When learning, we should get information with our own way and solve problems through our own understanding.
Learning is to accumulate after thinking carefully.
For me, reading a book several times will have different experiences and gains.
When listening to experts, we should have our own ideas.
When learning, we should combine our newly learned knowledge with our existing knowledge.
“Learned” means understanding and making it into ours.

#### Speed of Learning

When I can’t learn something quickly, I will give it up.^∗^
I can never solve the problem, which I think cannot be solved at first time.^∗^
Knowledge that cannot be understood quickly may not be understood well in the future.^∗^
It is a waste of time to delve into problems for which definite answers are impossible to obtain.^∗^
It takes a long time to solve difficult problems, and only truly intelligent people will be rewarded.^∗^
Facing new things, I know whether I can learn well or not in the first few minutes.^∗^

#### Ability to Learn

Ability is innate, no matter how hard you try, it is difficult to change.^∗^
Students who learn well are naturally more intelligent.^∗^
Some people are born with strong learning abilities, while others have limited learning abilities. ^∗^
Students who are really smart will get good grades even without working hard.^∗^
The potential for learning is inherent. ^∗^
Some people are born smart, while others are born retarded.^∗^

#### Value of Learning

Learning is fun and enjoyable.
I believe that knowledge changes destiny.
Learning often annoys me.^∗^
Reading is a pleasant thing for me.
I can often feel happy in my learning.
I enjoy doing research.
I am willing to delve into things that I do not understand.
I think doing research and studying are boring.^∗^
Note: Asterisks for reverse items.
